# Supplementary material for: The neural basis of attentional selection in goal-directed memory retrieval
Source: Sci Rep. 2024 Sep 9;14:20937. doi: 10.1038/s41598-024-71691-x (PMC11385572; doi:10.1038/s41598-024-71691-x)
Supplement: Supplementary file 1 — Supplementary Information. [file 41598_2024_71691_MOESM1_ESM.docx]

**Supplementary materials**

**The Neural Basis of Attentional Selection in Goal-Directed Memory Retrieval**

Melinda Sabo, Edmund Wascher, Daniel Schneider

To exclude the possibility that the decoding results reported in the manuscript are confounded by the mouse click in the Cueing phase, we conducted an additional decoding analysis. This was necessary due to differences in response times between the selective and neutral cue conditions: participants were faster in internally retrieving the relevant location(s) in the selective cue condition (M = 2239.93 ms, SD = 1068.72 ms) compared to the neutral cue condition (M = 2584.75 ms, SD = 1309.62 ms), with a statistically significant difference: t(29) = -3.66, *p* < .001.


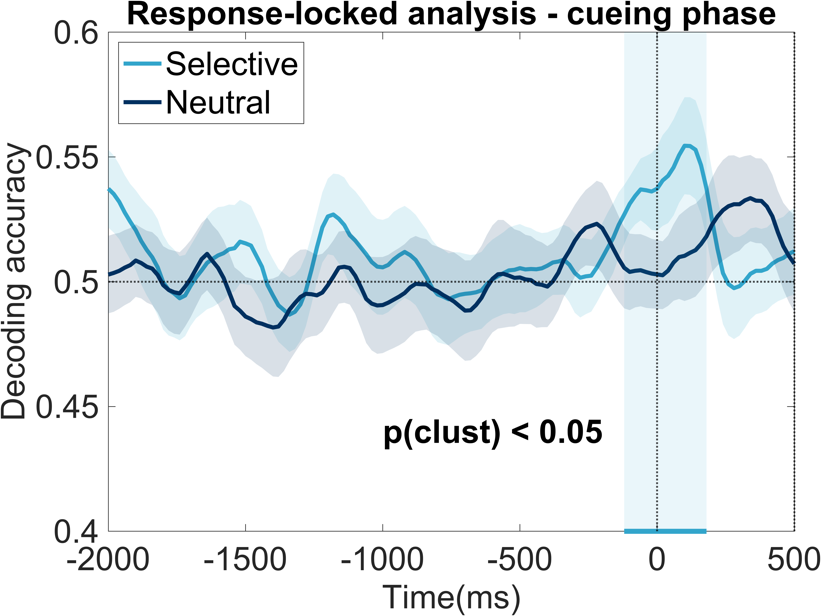
 We applied the same cluster-based permutation statistics within the -1500 to 500 ms time window around the response. We compared decoding accuracy in both conditions against chance level (i.e., 0.5). As Figure S1 indicates, the results revealed a significant cluster in the selective cue condition (-120 to 180 ms), but not in the neutral cue condition.

**Figure S1.** Decoding analysis (8-13 Hz activity) – response-locked trials of the Cueing phase. The figure shows the average decoding accuracy over time separately for the selective and neutral cue conditions. The light blue shaded area shows the significant cluster obtained when decoding accuracy in the selective cue condition is contrasted to chance level (significant cluster: -120 to 180 ms). The shaded area around the decoding accuracy time series depicts the standard error of the mean. The dotted vertical line at time point 0 indicates mouse clicks in the Cueing phase.

This result shows that the mouse click does not confound the analysis reported in the manuscript. If it did, we would have observed a similar decoding accuracy pattern in both conditions. Second, the pattern depicted in the figure partially replicates our cue-locked analysis reported in the manuscript, indicating significant above-chance decoding for the selective cue condition, but not for the neutral cue condition. In our view, the significant cluster in the selective cue condition might reflect that right before participants click to finalize internal retrieval, the information is reactivated or attended. This interpretation aligns with the pattern found by Kerren et al. (2019), who observed better decoding of retrieved information in a response-locked compared to a stimulus-locked analysis. However, we note that in the previous study, information reactivation occurred earlier, before the response, whereas in our case, it coincides more closely with the response.

**References**

Kerrén, C., van Bree, S., Griffiths, B. J., & Wimber, M. (2022). Phase separation of competing memories along the human hippocampal theta rhythm. *eLife*, *11*, e80633.  https://doi.org/10.7554/eLife.80633
